# Digitale Souveränität in soziotechnischen Systemen – KI-Nutzung und Krisenbewältigung

**DOI:** 10.1007/s11612-023-00674-9

**Published:** 2023-03-30

**Authors:** Annelie Hofmann, Ernst A. Hartmann, Alexandra Shajek

**Affiliations:** Institut für Innovation und Technik (iit) Berlin, Berlin, Deutschland

**Keywords:** Soziotechnische Systeme, Digitale Souveränität, Partizipative Methoden, Sociotechnical systems, Digital sovereignty, Participatory methods

## Abstract

Dieser Beitrag der Zeitschrift *Gruppe. Interaktion. Organisation*, diskutiert das Zusammenspiel der Konzepte soziotechnische Systeme und digitale Souveränität. Die jeweiligen theoretischen Hintergründe werden betrachtet und funktionale Beziehungen zwischen den beiden Gegenstandsbereichen hergeleitet. Es wird ein soziotechnischer Ansatz vorgeschlagen, mit dem Bewertungs- und Gestaltungskriterien für Handlungskontexte abgeleitet werden können, die der digitalen Souveränität von Unternehmen und Beschäftigten zuträglich sind. Vor diesem Hintergrund werden im Artikel zwei empirische Analyse- und Gestaltungsfelder skizziert: Im ersten empirischen Feld wird der Ansatz zur partizipativen Gestaltung von industriellen Arbeitsumgebungen eingesetzt, die durch die Einführung fortgeschrittener algorithmischer Systeme bzw. KI-Systemen (Künstliche Intelligenz) neu geprägt werden. Solche KI-basierten Systeme stellen u. A. wegen ihrer inhärenten Intransparenz eine große Herausforderung für Gestaltungen im Sinne digitaler Souveränität dar. In einem Workshop mit 50 Teilnehmenden aus verschiedenen Wirtschaftsunternehmen und Forschungseinrichtungen wurde dazu eine Matrix zur soziotechnischen Analyse von Arbeitsumgebungen entlang realer Fallbeispiele angewendet. Die drei Fallbeispiele stammten aus den Anwendungsfeldern Automobil, Brauerei und Verpackungstechnik. In allen drei Fällen geht es um die Integration von KI-gestützten Methoden in bestehende Arbeitsabläufe. Die Methode erlaubt eine strukturierte Interaktion mit synchroner Visualisierung von Gestaltungsideen, die für co-kreative Formate förderlich ist. Das zweite empirische Anwendungsfeld, auf das der soziotechnische Ansatz angewendet wurde, fokussiert die digitale Souveränität auf der Organisationsebene von Unternehmen und Einrichtungen. In der betrachteten Studie wurde erforscht, wie Organisationen mit den Herausforderungen der COVID-19-Pandemie umgegangen sind. Analysiert wurden fünf Branchen (Einzelhandel, Gesundheitswirtschaft einschließlich Pflege, Öffentliche Verwaltung, Unternehmensnahe Dienstleistungen, Öffentlicher Personennahverkehr) mit je vier Fallstudien, die die Auswirkungen der Krise auf der Grundlage leitfadengestützter Interviews sowohl mit der Arbeitgeber- als auch mit der Arbeitnehmerseite abbilden. Dabei zeigt sich, dass Zusammenhänge zwischen den Merkmalen soziotechnischer Systeme und digitaler Souveränität auch hier Interpretationshilfen bieten.

## Abstract (en)

Our contribution to the journal *Group. Interaction. Organization*, discusses the interplay between the concepts of sociotechnical system and digital sovereignty. The respective theoretical backgrounds are considered and functional relationships between the two subject areas are derived. A sociotechnical approach is proposed that can be used to develop evaluation and design criteria for contexts of action that are conducive to the digital sovereignty of companies and employees. Against this background, the article outlines two empirical fields of analysis and design: In the first empirical field, the approach is applied to the participatory design of industrial work environments that are being reshaped by the introduction of advanced algorithmic systems or AI (artificial intelligence) systems. Such AI-based systems pose a major challenge for design in terms of digital sovereignty, among other things because of their inherent opacity. In a workshop with 50 participants from various business enterprises and research institutions, a matrix for the socio-technical analysis of work environments was applied along real case studies. The three case studies came from the application fields of automotive, brewery and packaging technology. All three cases involve the integration of AI-supported methods into existing workflows. The method allows structured interaction with synchronous visualization of design ideas, which is conducive to co-creative formats. The second empirical application field to which the socio-technical approach was applied focuses on digital sovereignty at the organizational level of companies and institutions. The study under consideration explored how organizations dealt with the challenges of the COVID-19 pandemic. Five industries were analyzed (retail, health care including nursing, public administration, business-related services, public transport), each with four case studies mapping the impact of the crisis based on guided interviews with both the employer and employee sides. This shows that correlations between the characteristics of socio-technical systems and digital sovereignty also offer interpretative aids here.

## Einleitung

Digitale Souveränität bezeichnet die Möglichkeit und Fähigkeit unterschiedlicher Subjekte, sich digitale Technologien für ihre eigenen Zwecke nutzbar zu machen und in einer zunehmend digitalisierten Welt durch kompetentes Handeln eigene Ziele erfolgreich zu verfolgen. Sie kann auf unterschiedliche kollektive und individuelle Subjekte bezogen werden, wie etwa Staaten und Bürger:innen (Couture und Toupin 2019). Im Kontext der Wirtschaft können die individuellen Mitarbeiter:innen sowie das Unternehmen als Ganzes als solche Subjekte der digitalen Souveränität betrachtet werden (Hartmann 2021a, 2022).

Auf beiden Ebenen stellen sich die Herausforderungen spezifisch dar: Auf der Ebene des Individuums stellen sich Fragen der humanzentrierten, soziotechnisch begründeten Organisations- und Technikgestaltung neu, allerdings auf erheblich fortgeschrittenem technologischem Niveau. So ist etwa die Forderung nach transparenten, erklärbaren Systemen mit Künstlicher Intelligenz (KI) insofern besonders herausfordernd, als KI-Systeme inhärent intransparent sind und sich einfachen Erklärungen ihres Verhaltens entziehen – zumindest wenn sie auf maschinellem Lernen basieren. Transparenz kann allerdings indirekt etwa durch Approximation des Verhaltens des Systems und Darstellung dieser Approximation in sprachlicher oder visueller Form gegenüber den Nutzenden angestrebt werden. Aktuell werden erfolgversprechende Ansätze erklärbarer KI entwickelt (Molnar 2018; Wirth et al. 2021; Kraus et al. 2021; Pentenrieder et al. 2022).

Auf der Ebene des Unternehmens sehen sich Entscheidungsträger:innen mit Entwicklungen konfrontiert, die gleichzeitig Chancen und Risiken mit sich bringen. Dies betrifft einerseits die Beherrschung neuer Informationstechnologien für eigene Anwendungen, andererseits auch Geschäftsbeziehungen zu Anbietern digitaler Plattformen, die neue Geschäftsmodelle ermöglichen, aber auch Abhängigkeiten und Kompetenzverluste mit sich bringen können (Lehmann und Dörr 2022). Für beide Subjektebenen soll im Folgenden gezeigt werden, dass sich aus einem soziotechnischen Ansatz Bewertungs- und Gestaltungskriterien für Handlungskontexte ableiten lassen, die der digitalen Souveränität von Unternehmen und Beschäftigten zuträglich sind.

## Theoretische Hintergründe

### Vorbemerkung

 Im vorliegenden Beitrag werden theoretische Überlegungen zur digitalen Souveränität, die an anderer Stelle bereits publiziert wurden (Hartmann 2021a, 2022), angewendet auf zwei praktische Analyse- und Gestaltungskontexte. Der erste dieser Kontexte ist die Gestaltung von industriellen Arbeitssystemen unter Einsatz von algorithmischen bzw. KI-basierten Systemen (s. unten, Abschn. 3). Während dieser Kontext einen engen Bezug zur Gestaltung einzelner Arbeitsplätze und -systeme aufweist, bezieht sich der zweite Kontext auf die Ebene des Gesamtunternehmens; konkret geht es um die Bewältigung der Covid-19-Pandemie durch Unternehmen (s. unten, Abschn. 4).

In den folgenden Abschn. 2.2 und 2.3 werden diese Vorüberlegungen in sehr enger Anlehnung an die erwähnten Vorpublikationen (Hartmann 2021a, 2022) dargestellt. Es werden hier keine über diese Vorveröffentlichungen hinausgehenden Erwägungen angestellt, es geht vielmehr ausschließlich darum, den Leser:innen eine Einordnung der Anwendungsfälle zu ermöglichen, ohne zunächst die Vorveröffentlichungen lesen zu müssen.

### Soziotechnische Systeme

In der Theoriebildung zu soziotechnischen Systemen lassen sich hinsichtlich der Struktur solcher Systeme zwei Diskurskontexte erkennen. Beiden gemeinsam ist die Orientierung an Werten der Demokratie (vgl. Emery und Thorsrud 1982) und der humanzentrierten Technikgestaltung (Mumford 2006). Zunächst zu nennen ist die ‚ursprüngliche‘ auf das *Tavistock Institute of Human Relations* zurückgehende Denkrichtung (Trist und Bamforth 1951; Mumford 2006; Cherns 1976). Kernpostulat ist die gemeinsame Gestaltung des technischen und des sozialen Teilsystems – ‚the joint optimization of the social and technical systems‘ (Mumford 2006, S. 321). Dadurch wird eine zweigliedrige Struktur soziotechnischer Systeme unterstellt, mit sozialem und technischem Teilsystem.

Im Unterschied dazu besteht eine andere, eng mit Eberhard Ulich und dem damaligen arbeitspsychologischen Institut der ETH Zürich verbundene Tradition der Auseinandersetzung mit soziotechnischen Systemen (Ulich 2013; Strohm und Ulich 1997). Hier wurden grundlegende Theoreme und Ergebnisse der Tavistock-Tradition aufgenommen, zusätzlich gingen Aspekte der Handlungsregulationstheorie aus der Dresdner Schule der Arbeitspsychologie ein (Hacker und Richter 1990; Hacker 2005). Im Unterschied zur engeren Tavistock-Traditionslinie werden allerdings drei Teilsysteme des soziotechnischen Systems unterschieden: Mensch, Technik und Organisation (MTO) – dies zeigt sich besonders deutlich in der entsprechend benannten MTO-Analyse für die arbeitspsychologische Bewertung von Unternehmen (Strohm und Ulich 1997).

Durch die Unterscheidung von Menschen und Organisationen als zwei Arten von handelnden Akteuren können weitere Aspekte in die Analyse und Gestaltung soziotechnischer Systeme aufgenommen werden, wie etwa das Phänomen des organisationalen Lernens, also das Lernen der Organisation selbst als sozialer Entität (Argyris und Schön 1996; unter dem Aspekt soziotechnischer Systeme auch Hartmann 2005).

### Soziotechnische Systeme und digitale Souveränität

Im iit-Projekt ‚Digitale Souveränität in der Wirtschaft‘ werden Mitarbeiter:innen und Unternehmen als die beiden relevanten Subjektebenen digitaler Souveränität betrachtet (Hartmann 2021a, 2022). Im Kontext dieses Projekts wird Bezug genommen auf soziotechnische Analyse- und Gestaltungsansätze, insofern orientiert an der ‚Zürcher Schule‘, als auch hier die drei Teilsysteme Mensch, Technik und Organisation angenommen werden (Hartmann 2021b). Weiterhin werden handlungs- und kontrolltheoretische Konzepte herangezogen, hier insbesondere die Arbeiten von Rainer Oesterreich (1981), der Handlungsregulations- und Kontrolltheorien verbindet. Die enge Beziehung zum Konstrukt der digitalen Souveränität besteht darin, dass Konzepte wie Unabhängigkeit, Autonomie und Kontrolle – im Sinne einer Kontrolle der Menschen oder Organisationen über ihre Umweltbedingungen – sehr eng mit der Kernbedeutung digitaler Souveränität verbunden sind (Couture und Toupin 2019)^1^. Kontrolle in diesem Sinne ist somit ein Kernaspekt digitaler Souveränität.

Oesterreich (1981) unterscheidet zwei Aspekte von Kontrolle:

- Die *Effizienz* definiert als Wahrscheinlichkeit, mit bestimmten Handlungen bestimmte Ziele erreichen zu können; es besteht eine Nähe zum Konstrukt der Handlungssicherheit (z. B. Rau 1996).
- Die *Divergenz* im Sinne der Verfügbarkeit mehrere Handlungswege zu mehreren Zielen; hier besteht eine Nähe zu den Handlungsspielräumen und Freiheitsgraden (Hacker 2005; Osterloh 1983).

Er bietet formale Kenngrößen an für Effizienz, Divergenz und ein verbindendes Konstrukt, Effizienz-Divergenz (Oesterreich 1981; vgl. auch Hartmann 2021b). Die Konstrukte Effizienz, Divergenz und Effizienz-Divergenz stehen somit in Beziehungen zu etablierten Konzepten der Arbeits- und Organisationspsychologie wie Handlungsspielräume und Freiheitsgrade (z. B. Hacker 2005) sowie Handlungssicherheit (z. B. Rau 1996; Osterloh 1983), wie oben dargestellt. Ein wesentlicher Vorzug des Ansatzes von Oesterreich (1981) besteht einerseits darin, diese ansonsten unverbundenen Konzepte in einen konsistenten theoretischen Rahmen integriert zu haben. Andererseits lassen sich, im Kontext praktischer Analyse und Gestaltung soziotechnischer Systeme, prägnante Merkmale technischer Systeme wie Zuverlässigkeit, Robustheit und Resilienz der Effizienz und Merkmale wie flexible Benutzerführung und Adaptierbarkeit der Divergenz^2^ zuordnen.

Die Kombination der drei Teilsysteme des soziotechnischen Systems mit den Aspekten der handlungstheoretischen Kontrolle – Effizienz, Divergenz und, als Vorbedingung, Transparenz – führt zu einer 3 × 3-Matrix der Gestaltungsgegenstände digitaler Souveränität, zunächst auf der Ebene des Individuums (Tab. 1; Hartmann 2021b). Diese Tabelle wird im Folgenden zeilenweise besprochen.

**Table 1:** 

Aspekt der Kontrolle	Mensch	Technik	Organisation
Übergeordnet/Voraussetzung	Digitales Grundwissen/Digital Literacy	Transparenz/Erklärbarkeit	Transparenz über Aufgaben und Entscheidungsbefugnis
Effizienz	Aufgabenbezogenes (digitales) Spezialwissen	Technische Zuverlässigkeit, Robustheit, Resilienz	Aufgabenteilung und -kombination, soziale Unterstützung
Divergenz	Interdisziplinäres (digitales) Spezialwissen, Kompetenzen als Selbstorganisationsdispositionen	Eingriffsmöglichkeiten in das System auf wählbaren Regulationsebenen	Entscheidungs‑, Tätigkeits‑, Handlungsspielräume

Entscheidend für das Kontrollerleben sind die vom Menschen wahrnehmbaren und wahrgenommenen Kontrollmöglichkeiten. Eine in diesem Sinne elementare Voraussetzung im Bereich des Teilsystems Mensch ist eine digitale Grundbildung, eine elementare Digital Literacy. Die Transparenz im Bereich des technischen Teilsystems ist aktuell Gegenstand intensiver Diskussion, vor allem im Hinblick auf die Erklärbarkeit von KI-Systemen (Kraus et al. 2021; Pentenrieder et al. 2022). Seitens des Teilsystems Organisation ist eine wesentliche Voraussetzung für das Kontrollerleben eine grundsätzliche Transparenz über die dem Individuum zugewiesenen Aufgaben und über seine Entscheidungsbefugnis.

Für den Aspekt der *Effizienz* benötigt der Mensch ein hinreichendes Fachwissen, um sich in digitalen Handlungsfeldern sicher bewegen zu können. Seitens des Teilsystems Organisation wird durch die grundsätzliche Aufgabenteilung und -kombination bestimmt, inwieweit die jeweiligen Arbeitsaufgaben überhaupt von Menschen auf hohem und nachhaltigem Leistungsniveau bearbeitet werden können (vgl. das Konzept der vollständigen Tätigkeiten, Hacker 2005; Hacker und Richter 1990). Für das technische Teilsystem stehen unter dem Aspekt der Effizienz Merkmale wie technische Zuverlässigkeit, Robustheit und Resilienz der technischen Systeme im Vordergrund.

Bezüglich der *Divergenz* ist ein wesentliches Merkmal des Menschen das Vorhandensein kognitiver Ressourcen, die nicht nur die Bewältigung immer gleicher Anforderungen erlauben, sondern auch in neuen, ungewohnten, dynamischen Situationen Handlungsfähigkeit ermöglichen. Neben interdisziplinärem Wissen sind dies insbesondere Kompetenzen als Selbstorganisationsdispositionen im von John Erpenbeck definierten Sinn (Erpenbeck et al. 2017). Hinsichtlich des organisationalen Teilsystems sind Freiheitsgrade und Handlungsspielräume Voraussetzungen von Divergenz (Osterloh 1983; Hacker 2005). Für das Teilsystem Technik dient insbesondere das Ecological Interface Design (EID) der Divergenz (Vicente und Rasmussen 1992). Dieser Gestaltungsansatz beruht auf der Vorstellung hierarchisch aufgebauter Handlungskontrolle bzw. -regulation. Dabei wird von hohen, intellektuellen Ebenen der Handlungsregulation bis zu niedrigeren, sensumotorischen unterschieden (Hacker 2005). Der EID-Ansatz läuft darauf hinaus, komplexe Mensch-Maschine-Schnittstellen so zu gestalten, dass die Nutzenden möglichst zu jeder Zeit die Möglichkeit haben, wahlweise auf jeder dieser Handlungsregulationsebenen in das System einzugreifen.

Diese soziotechnischen, an der digitalen Souveränität orientierten Gestaltungsprinzipien sind eine Grundlage der im nächsten Abschnitt dargestellten ko-kreativen Methode zur Gestaltung KI-basierter Arbeitsumgebungen.

Tab. 2 zeigt Elemente der soziodigitalen Souveränität auf der Ebene der Organisation. Die Zeilen der Tabelle entsprechen wie oben im Fall der Individuen den beiden Aspekten der Kontrolle, also Effizienz und Divergenz. In den Spalten finden sich die aus der Wissensbilanzierung bekannten drei Kapitalarten Human‑, Struktur- und Beziehungskapital (Mertins 2005). Der Verwendung dieser Konstrukte im Kontext der digitalen Souveränität liegt die Annahme zugrunde, dass für die digitale Souveränität des Unternehmens die Ausprägungen dieser drei Kapitalarten als zentrale Ressourcen der Souveränität in digitalen Transformationsprozessen wesentlich sind. Ursprünglich (Hartmann 2021b) stellte dies eine reine Hypothese dar. Auf Basis dieser Annahme wird im Zuge des vorliegenden Textes untersucht, inwieweit sich in realen Krisenbewältigungsprozessen in Unternehmen solche Aspekte, insbesondere hinsichtlich des organisationalen Lernens (s. unten), finden lassen (vgl. Abschn. 4).

**Table 2:** 

Aspekt der Kontrolle	Humankapital	Strukturkapital	Beziehungskapital
Effizienz	Tiefe des (digitalen) Fachwissens	Single-Loop-Lernen	Verlässlichkeit der (digitalen) Dienstleistungsbeziehungen
Divergenz	Vielfalt des (digitalen) Fachwissens	Double-Loop-Lernen	Handlungsspielräume innerhalb der (digitalen) Dienstleistungsbeziehungen

Human- und Strukturkapital spiegeln die oben für die Individuen in den Kategorien Mensch und Organisation abgebildeten Sachverhalte aus der Perspektive des Unternehmens bzw. der Organisation. Humankapital bezeichnet das für die Organisation verfügbare Wissen der Beschäftigten. Im Hinblick auf die Effizienz ist dabei eher die Tiefe des (digitalen) Fachwissens bedeutsam, für die Divergenz ist es eher die Vielfalt dieses Wissens.

Das Strukturkapital beschreibt Potenziale der internen Strukturen der Organisation unter den Aspekten der Lern- und Innovationspotenziale. Dabei kommen Modelle des organisationalen Lernens zum Tragen (Argyris und Schön 1996)^3^. Effizienz wird aufgebaut durch Single-Loop-Lernen, das fortlaufende Verbessern im Unternehmen etablierter Prozesse. Divergenz entsteht durch Double-Loop-Lernen, das Entwickeln neuer organisationaler Prozesse und Strukturen im Unternehmen.

Beziehungskapital schließlich betrifft die Beziehungen des Unternehmens zu externen Akteuren. Im Kontext der digitalen Souveränität sind hier besonders IT-Dienstleister von Interesse, etwa Anbieter von Cloud- und Plattformdiensten, im Hinblick auf die Verlässlichkeit dieser Dienstleistungsbeziehungen (Effizienz) und die Handlungsspielräume innerhalb dieser Dienstleistungsbeziehungen (Divergenz).

Diese Aspekte der soziotechnischen Gestaltung im Hinblick auf digitale Souveränität werden weiter unten (Abschn. 4) herangezogen zur Interpretation der empirischen Ergebnisse aus der Begleitforschung zur Bewältigung der Folgen der Covid-19-Pandemie durch deutsche Unternehmen und Einrichtungen.

### Zwischenfazit und Forschungsfragen

In Vorveröffentlichungen wurde ein theoretisches Konstrukt zur digitalen Souveränität in der Wirtschaft, bezogen auf ganze Unternehmen und einzelne Beschäftigte, vorgestellt (Hartmann 2021a, 2022).

Das Konstrukt enthält Aspekte der digitalen Souveränität auf der Ebene einzelner Arbeitssysteme (neun Aspekte in einer 3 × 3-Matrix) und auf der Ebene des Gesamtunternehmens (sechs Aspekte in einer 2 × 3-Matrix^4^). Die Forschungsfragen lauten nun:

- Wie kann das Konstrukt der digitalen Souveränität – insbesondere das auf der Ebene einzelner Arbeitssysteme formulierte – dazu beitragen, in einem interaktiven Gestaltungsworkshop wesentliche Aspekte der Gestaltung KI-gestützter Arbeitssysteme in ihrem Zusammenhang zu erkennen?
- Wie kann das Konstrukt der digitalen Souveränität – insbesondere das auf der Ebene der Unternehmen formulierte – dazu beitragen, Aspekte der Krisenbewältigung von Unternehmen – am Beispiel der Covid-19-Pandemie – in ihrem Zusammenhang zu erkennen?

## Inklusive, partizipative Gestaltung digitaler Systeme zur Gewährleistung von digitaler Souveränität der Facharbeiter:innen

### Workshopdesign und -durchführung^5^

Die Prinzipien der Analyse und Gestaltung soziotechnischer Systeme unter dem Aspekt der digitalen Souveränität wurden in einem Workshop auf ihre Anwendbarkeit in realen Gestaltungskontexten untersucht. Der Bedarf an Instrumenten wie diesem Workshop wurde im Rahmen von Arbeitsplatzstudien bei kleinen und mittelständischen Unternehmen im Werkzeug- und Formenbau identifiziert. Insbesondere wurde deutlich, dass es den Unternehmen schwerfällt, die Implikationen des Einsatzes von KI-gestützten Systemen für die konkrete betriebliche Situation abzuschätzen und Ansatzpunkte für eine anwendungsspezifische Gestaltung zu identifizieren (Pentenrieder und Hartmann 2022). Im Workshop wurde erprobt, wie Beteiligungsformate aussehen sollten, die Personen mit unterschiedlichen fachlichen Hintergründen einen niedrigschwelligen Zugang zur Gestaltung ihrer eigenen Technologien am Arbeitsplatz ermöglichen können (in Anlehnung an die Forschungstradition des Participatory Design, Simonsen und Robertson 2013). Im Fokus standen Systeme, die im industriellen Arbeitsalltag genutzt werden und mit Systembestandteilen ausgestattet sind, die auf KI basieren. Das bedeutet, dass diese Systeme mit dynamischen Algorithmen, statistischen Verfahren und großen Datenmengen arbeiten. Eine logische Nachvollziehbarkeit ist hierbei nur schwer zu gewährleisten. Es wurden drei reale Anwendungsszenarien mit entsprechenden KI-Systemen betrachtet – aus der Automobilindustrie, dem Brauereiwesen und der Verpackungstechnik. Konkret geht es um folgende Systeme:

- ein System zur Kalibrierung des Schließmechanismus einer Heck- oder Kofferraumklappe eines PKW,
- ein System zur Analyse und Optimierung von geschmacksrelevanten Faktoren (wie Rezeptur/Zutaten und Prozessparametern) im Bierbrauprozess,
- ein System zur Unterstützung der Einrichtung und Wartung von Abfüllanlagen für Stoffe unterschiedlicher Viskosität (z. B. verschiedene Kosmetika).

Der Workshop hatte zwei Ziele: Einerseits sollte eine zeitgemäße Austauschmöglichkeit erprobt werden, in der Personen mit unterschiedlichen Bildungs- und Erfahrungshintergründen interdisziplinär darüber diskutieren können, was Erklär- und Kontrollierbarkeit für unterschiedliche Kreise von Anwendenden bedeutet und wie Erklärungen für komplexe Systeme aussehen könnten. Andererseits sollten Gestaltungsmerkmale für KI-gestützte soziotechnische Systeme, wie sie aus theoretischen Überlegungen abgeleitet wurden, durch die Gruppendiskussionen anhand konkreter Fälle validiert und konkretisiert werden.

**Figure 1:**
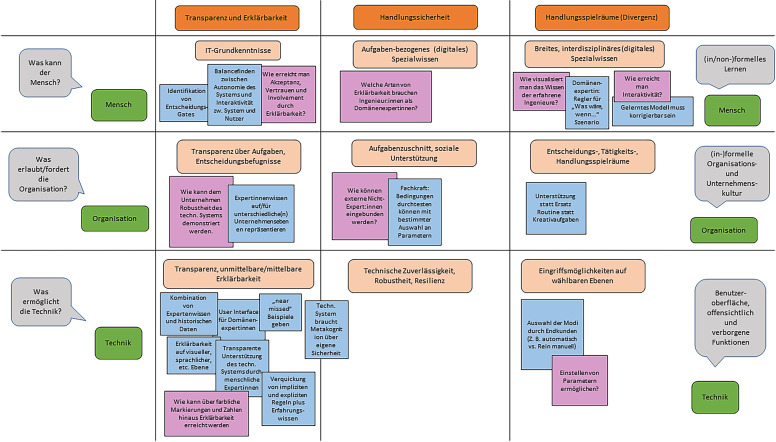

Im Workshop wurden die oben (Abschn. 2.2) genannten soziotechnischen Analyse- und Gestaltungsprinzipien zu einer Methode operationalisiert. Über die soziotechnische Matrix (siehe Tab. 1) konnten die Teilnehmenden ihre Ideen und Anmerkungen bestimmten Aspekten zuordnen. Die Matrix wurde über eine Kollaborationsplattform geteilt (siehe Abb. 1). Sie ermöglichte, dass die Teilnehmenden vielschichtige Ideen und Erfahrungen in die technische Systemgestaltung einbringen konnten.

An dem Workshop, der am 1. Dezember 2021 online stattfand, nahmen insgesamt 50 Personen aus unterschiedlichen Wirtschaftsbetrieben und Forschungseinrichtungen teil. Die Unternehmen stammten aus den Branchen Automobil, Maschinenbau sowie Mess- und Steuerungstechnik. Unter den wissenschaftlichen Einrichtungen waren Universitäten ebenso vertreten wie außerhochschulische Forschungseinrichtungen, darunter Institute der Fraunhofer-Gesellschaft. Zu den fachlichen Hintergründen der Beteiligten gehörten technisch-naturwissenschaftliche ebenso wie sozial- und wirtschaftswissenschaftliche Qualifikationen. Alle 50 Personen nahmen zunächst an einem einleitenden Veranstaltungsblock teil, in dem die theoretischen Grundlagen der soziotechnischen Matrix und die drei Anwendungskontexte vorgestellt und diskutiert wurden. Anschließend teilten sich die Teilnehmenden in drei Teilgruppen auf, die jeweils einen der Anwendungsfälle bearbeiteten. Diese Diskussionen in den Kleingruppen wurden von Zeichner:innen begleitet und dokumentiert. In einer abschließenden Workshop-Phase stellten die drei Gruppen ihre Ergebnisse – Einträge in die Matrix und zeichnerisch dokumentierte Analyseergebnisse sowie Gestaltungsideen für Mensch-Maschine-Schnittstellen – im Plenum vor und diskutierten sie mit den anderen Teilnehmenden.

**Figure 2:**
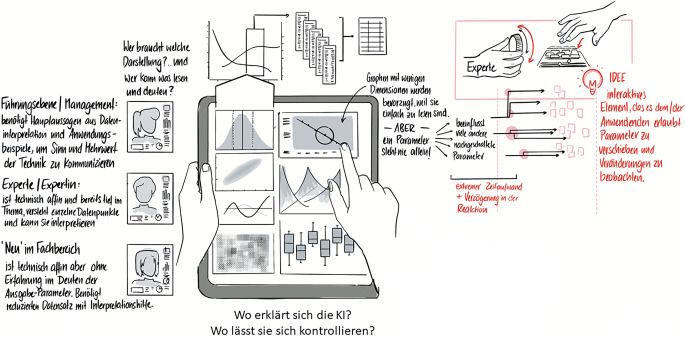

Mithilfe der soziotechnischen Analyse kann in der Konzeption der neuen Technik bedacht werden, dass die sozialen Bedürfnisse unterschiedlicher Gruppen von Nutzenden in die Gestaltung technischer Systeme einfließen (z. B. Gestaltung der Benutzeroberflächen). Jede Gruppe stellt andere Fragen an ein technisches System, dementsprechend muss stets die Frage vorangestellt werden, für wen eine Technologie sich erklären muss. Es ist ein Unterschied, ob eine Information an das Management, an einen Auszubildenden oder an eine versierte Fachkraft gerichtet ist (siehe Abb. 2).

### Ergebnisse und Zwischenfazit

Die Leitfragen, die sich aus den Gestaltungsaspekten der soziotechnischen Matrix ergeben, erwiesen sich als gut geeignet, die Diskussion zu den Anwendungsfällen zu stimulieren und zu strukturieren. Einerseits wurden alle neun Felder (vgl. Tab. 1) tatsächlich genutzt, andererseits ließen sich alle vorgebrachten Aspekte recht eindeutig zuordnen. Die von den Teilnehmer:innen genannten Aspekte konkretisieren und vertiefen die in den einzelnen Zellen dargestellten übergeordneten Aspekte.

Durch die Begleitung professioneller Zeichner:innen konnten sich die Beteiligten kontinuierlich ein „Live-Bild davon machen“, wie ihre eigenen Anforderungen zu denen der anderen Teilnehmenden passen, und welche Aspekte einer genaueren Betrachtung bedürfen. Diese Methode ermöglichte eine anschauliche und niedrigschwellige Diskussion über die Techniksysteme.

Gleichzeitig hört die soziale Einbindung eines technischen Systems nicht an der Benutzeroberfläche eines Systems auf. Auch die Organisationsebene muss in die Gestaltung eines Systems einbezogen werden, damit eine neue Software gebrauchstauglich in bewährte Arbeitsprozesse integriert werden kann. Ebenso müssen Schulungen eingeplant werden und für Beteiligungsformate finanzielle Ressourcen vorgehalten werden. Damit sollte das Unternehmen frühzeitig soziotechnische Gestaltungsmöglichkeiten in der Entwicklung einer neuen Software einplanen.

Eine weitere wichtige Erkenntnis des Workshops war, dass Erklärungen nicht unbedingt von denen formuliert werden sollten, die die Systeme auch technisch gestalten. Denn es benötigt eine Vermittlungsebene, die diese technischen Aspekte in die Sprache unterschiedlicher Kreise von Anwendenden übersetzt und deren soziale Bedarfe berücksichtigt. Nur so kann eine nachvollziehbare Technik gestaltet werden^6^, die auch verschiedene Gruppen von Nutzenden überzeugen und abholen kann.

Gerade solche partizipativen Elemente wie die gemeinsame Befüllung der soziotechnischen Matrix und die gemeinsame Gestaltung der Visualisierung einer Benutzeroberfläche haben das Potenzial, auch technisch nicht affine Personen in die Technikgestaltung zu integrieren. Da diese Personen häufig zentrale und gestaltungsweisende Fragen in die Softwareentwicklung einbringen können, wobei sie als Nutzende den technischen Hintergrund nicht kennen können, müssen sich Softwaregestaltende folglich damit beschäftigen, wie diese wertvollen Perspektiven gerade bei der Entwicklung komplexer Technologien eingeholt werden können.

## Digitale Souveränität am Beispiel der organisationalen Bewältigung der Corona-Krise durch Unternehmen

### Fallstudien zu den Auswirkungen der Corona-Pandemie auf betriebliche Veränderungsprozesse

Die im Folgenden präsentierten Ergebnisse entstammen einer Studie im Auftrag des Bundesministeriums für Arbeit und Soziales (BMAS) zu den Auswirkungen der Corona-Pandemie auf betriebliche Veränderungsprozesse (Busch-Heizmann et al. 2021; die folgenden Ausführungen basieren auf dieser Quelle). Die Untersuchung analysiert für fünf Branchen (Einzelhandel, Gesundheitswirtschaft einschließlich Pflege, öffentliche Verwaltung, unternehmensnahe Dienstleistungen, öffentlicher Personennahverkehr) mit je vier Fallstudien die Auswirkungen der Krise auf der Grundlage leitfadengestützter Interviews sowohl mit der Arbeitgebenden- als auch mit der Arbeitnehmendenseite im Winter 2020/21 und im Frühjahr 2021. Sie erscheint uns geeignet, da hiermit eine aktuelle Analyse der Auswirkungen des pandemiebedingten Digitalisierungsschubes in der Breite vorliegt. Im Folgenden sollen zunächst die Hauptergebnisse der Studie, wie sie bereits publiziert wurden, dargestellt werden, bevor sie dann noch einmal mit Blick auf das Konzept der digitalen Souveränität interpretiert werden.

Insgesamt zeigen die Fallstudien, dass es vielen Organisationen sowohl bei der Durchführung der Transformationsprozesse als auch in der Pandemie gelang, erfolgreich auf innovationsförderliche Ressourcen wie Kompetenzen, Organisationsstrukturen und externe Netzwerke zurückzugreifen (Busch-Heizmann et al. 2021).

Es zeigt sich, dass sich viele der Beschäftigten bereits zu Beginn der Pandemie relativ zügig die notwendigen Digitalkompetenzen aneignen, die sowohl hinsichtlich der Tiefe als auch der Vielfalt als ausreichend zur Pandemiebewältigung wahrgenommen werden (Effizienz und Divergenz, vgl. Tab. 2). Neben IT-Kenntnissen, wie sie zum Einsatz bestehender und neuer Software notwendig sind, wird deutlich, dass der Einsatz digitaler Tools insbesondere ein erhöhtes Ausmaß an sozialen Kompetenzen erfordert (Busch-Heizmann et al. 2021). Dies betrifft sowohl die Mitarbeiter:innen insgesamt, zum Beispiel bei der virtuellen Teamarbeit, insbesondere aber auch Führungskräfte, denn die Personalführung aus der Distanz wird als wesentlich anspruchsvoller eingestuft als die klassische Führung vor Ort (ebd.).

### Interpretation der Ergebnisse mit Blick auf digitale Souveränität

Die obigen Ausführungen lassen sich dahingehend interpretieren, dass soziale Kompetenzen als wichtige Voraussetzung für digitale Souveränität gelten können. Die Tatsache, dass die betrachteten Unternehmen und Einrichtungen über „etablierte und erprobte Strukturen zur kontinuierlichen Anpassung von Strategie und Arbeitsorganisation an Veränderungen im gesellschaftlichen und marktseitigen Umfeld“ (Busch-Heizmann et al., S. 108) verfügen, deutet darauf hin, dass dieses hohe Strukturkapital den Organisationen in der Krise Anpassungsprozesse sowohl im Sinne eines Single- als auch Double-Loop-Lernens erlaubt. Dabei dominierte in der ersten Phase der Pandemie in der Regel der „Überlebensmodus“, in dem überwiegend die Umstellung von Arbeitsprozessen von analog auf digital erfolgte, mit dem Ziel, eine Verzögerung von Arbeitsprozessen möglichst zu vermeiden (Busch-Heizmann et al. 2021). Die technische Ausstattung wurde fortlaufend verbessert und notwendige organisatorische Maßnahmen ergriffen. Nach anfänglicher Unsicherheit kam es zu einer erfolgreichen Anpassung von Prozessen (ebd.).

Insbesondere in der zweiten Pandemiephase lassen sich dann in vielen Unternehmen und Einrichtungen häufiger Double-Loop-Lernen-Prozesse beobachten, insofern, dass zu dieser Zeit die Optimierung der digitalen Organisation von Arbeit im Vordergrund stand. Dabei wurden viele Prozesse nicht einfach nur digitalisiert, sondern zunächst auf den Prüfstand gestellt, das heißt, es wurden Prozesse verschlankt, abgeschafft oder neue Softwaretools oder Apps eingesetzt (Busch-Heizmann et al. 2021). In einer Einrichtung des Gesundheitswesens wurde beispielsweise eine telefonische Terminerinnerung für Patient:innen eingeführt, die sich im weiteren Verlauf sehr bewährt hat und auch nach der Pandemie beibehalten werden soll (ebd.).

Im Ergebnis erscheint nahezu allen Interviewpartner:innen eine Rückkehr zum Status quo der Arbeitsorganisation vor der Pandemie als ausgeschlossen und auch nicht wünschenswert. Im Sinne eines erfolgreichen Double-Loop-Lernens hat sich vielerorts ein „New Normal“ etabliert (Busch-Heizmann et al. 2021). In der Pandemie erstmalig erprobte Maßnahmen wie digitale Schulungen stellen inzwischen neue Standards dar. Aber auch digitale Formen der Arbeitsorganisation werden als Teil eines neuen Regelbetriebes gesehen. Antizipiert werden langfristig hybride Formen mit alternierenden Phasen aus mobiler und Präsenzarbeit (ebd.). Es besteht Konsens, dass „durch die intensive Auseinandersetzung und Erprobung von digitalen Arbeitsformen (…) auch Voraussetzungen für eine souveräne Gestaltung künftiger Digitalisierungsprozesse verbessert“ (Busch-Heizmann et al. 2021, S. 79) wurden.

## Fazit

Es wurde ein Ansatz vorgestellt, der die Analyse und Gestaltung soziotechnischer Systeme auf der Ebene der Beschäftigten wie auch auf der Ebene der Unternehmensführung unter dem Aspekt der digitalen Souveränität erlaubt. Der soziotechnische Ansatz hatte folgende Wirksamkeit: Im Co-Creation-Workshop zeigte sich dieser Ansatz als hilfreiche Organisationsstruktur zur Anregung und Strukturierung von Diskussionen. Vor allem die Herstellung von Bezügen zwischen den Feldern „Mensch“, „Technik“ und „Organisation“ unterstützte die Diskussion einer Teilnehmer:innenschaft, die sehr interdisziplinär geprägt war. Von besonderer Bedeutung ist dieser methodische Ansatz des Co-Creation-Workshops für die menschengerechte Gestaltung soziotechnischer Systeme, insbesondere dann, wenn hochentwickelte algorithmische und KI-gestützte Systeme zum Einsatz kommen. Neben der soziotechnischen Analyse- und Gestaltungsmatrix erwies sich die Begleitung durch professionelle Zeichner:innen als sehr hilfreich – dadurch konnten konkrete Ideen der Teilnehmer:innen sofort visualisiert und für die weitere Diskussion sowie nicht zuletzt auch für die Dokumentation der Workshopergebnisse verwendet werden. Für die Zukunft wird eine Weiterentwicklung des Ansatzes zu einem Verfahren zur Auditierung und Zertifizierung von Anwendungen solcher Systeme unter dem Aspekt der digitalen Souveränität der Beschäftigten und des Unternehmens geplant (Pentenrieder et al. 2022).

Nicht nur als methodisches Werkzeug, sondern auch zur wissenschaftlichen Analyse empirischer Studien bewährt sich dieser Ansatz, wie eine Interpretation von Fallstudien zur Bewältigung der Covid-19-Pandemie zeigt. Mit diesem Ansatz konnte die Aneignung der Digitalkompetenzen in Krisenzeiten reflektiert und hinsichtlich der Tiefe als auch der Vielfalt differenziert werden. Dabei werden wiederum soziotechnische Aspekte sichtbar, wie etwa, dass neben IT-Kenntnissen gerade in Krisenzeiten ein erhöhtes Ausmaß an sozialen Kompetenzen sowie neben individuellem auch organisationales Lernen erforderlich sind.

Für die Zukunft sind weitere empirische Erprobungen vorgesehen, bei denen auch untersucht werden wird, welche Aspekte, die sich nicht aus den hier gewählten soziotechnischen, handlungs- und kontrolltheoretischen Prinzipien ableiten lassen, dennoch unter praktischen Aspekten berücksichtigt werden müssen. Eine weitere offene Frage bezieht sich auf das Verhältnis zwischen digitaler Souveränität und Resilienz. In der Diskussion empirischer Ergebnisse (vgl. Abschn. 4) wurde deutlich, dass insbesondere zwischen digitalen und sozialen Kompetenzen und dem organisationalen Lernen enge Bezüge bestehen. Diese Bezüge sollten in der Zukunft theoretisch analysiert und auf praktische Auswirkungen untersucht werden.

## Footnotes

1 Es wird in diesem Kontext gelegentlich auch von soziodigitaler Souveränität gesprochen (Stubbe 2017). In ähnlicher Weise könnte von soziodigitalen Systemen – im Unterschied zu soziotechnischen Systemen – gesprochen werden, wenn man etwa den Bezug auf digitale Technologien besonders betonen wollte. Dies wäre für den hier vorliegenden Beitrag durchaus angemessen. Andererseits gibt es zu dieser Terminologie nach Kenntnis der Autor:innen noch keine tiefere Diskussion in der Scientific Community, sodass hier am etablierten Begriff des soziotechnischen Systems festgehalten wird.

2 Zum Aspekt der Divergenz im Kontext der Gestaltung technischer Systeme vgl. auch weiter unten die Ausführungen zum Ecological Interface Design (EID, Vicente und Rasmussen 1992).

3 Der Zusammenhang zwischen dem Konzept des soziotechnischen Systems und dem des organisationalen Lernens ist sehr eng, da sich beide Ansätze auf systemtheoretische Konstrukte beziehen. Dies wird unmittelbar deutlich in den Begriffen single und double *loop* learning – der ‚loop‘ ist ein Regelkreis im systemtheoretischen bzw. kybernetischen Sinn. Dieser Zusammenhang wurde auch in der Literatur häufig thematisiert (z. B. Molleman und Broekhuis 2001; Hartmann 2005; Meyer 2005; Marquardt 2019).

4 Es könnte gefragt werden – und wurde auch gefragt –, inwieweit auf der Ebene der Organisation ebenfalls eine 3 × 3-Matrix entstehen könnte, indem, analog zur Ebene des Arbeitssystems, eine Zeile ‚Transparenz‘ ergänzt wird. Die Autor:innen sehen darin ein wertvolles Potenzial zur Weiterentwicklung des Konzepts. Bislang haben sie allerdings noch kein theoretisch gut begründetes und mit den anderen Elementen kompatibles theoretisches Konstrukt zur Beschreibung der (Umwelt‑) Transparenz aus Unternehmenssicht identifizieren können.

5 Das Vorgehen des Workshops wurde bereits in einem Artikel von Pentenrieder und Hartmann (2022) unter dem Aspekt erklärbare KI beschrieben.

6 Systeme des maschinellen Lernens wie etwa neuronale Netze sind insofern grundsätzlich intransparent und nicht nachvollziehbar, weil ab einer gewissen Komplexität des Netzes niemand nachvollziehen kann, wie genau die Informationsverarbeitung zwischen den einzelnen Neuronen abläuft – auch die Systementwickler:innen können dies nicht. Transparenz kann insofern hergestellt werden, als dass das Verhalten des Systems möglichst gut approximiert und für die Nutzenden dargestellt wird, etwa durch sprachliche Beschreibungen, Flussdiagramme oder andere Visualisierungen. In diesem letzteren Sinne ist hier die Gestaltung nachvollziehbarer Technik gemeint.
